# Optimization of Improved YOLOv8 for Precision Tomato Leaf Disease Detection in Sustainable Agriculture

**DOI:** 10.3390/s25051398

**Published:** 2025-02-25

**Authors:** Yue Shen, Zhaofeng Yang, Zohaib Khan, Hui Liu, Wenhua Chen, Shuyang Duan

**Affiliations:** 1 School of Electrical and Information Engineering, Jiangsu University, Zhenjiang 212013, China; shen@ujs.edu.cn (Y.S.); yangzhaofeng@stmail.ujs.edu.cn (Z.Y.); zai1318@foxmail.com (Z.K.); 2 Department of Aeronautical and Automotive Engineering, Loughborough University, Leicestershire, Loughborough LE11 3TU, UK; 3 Jiangsu Greenport Modern Agricultural Development Co., Ltd., Suqian 223800, China; duanshuyangsq@163.com

**Keywords:** sustainable agriculture, pesticide application, deep learning, tomato crop management, grouped depthwise convolutions

## Abstract

Increasing demand for sustainable agriculture necessitates precise and efficient crop management to minimize resource wastage and environmental impact. To improve the precision of pesticide application in tomato leaves, a real-time tomato leaf detection method using an improved YOLOv8 algorithm is proposed. The framework was developed by integrating Depthwise Grouped Convolutions and an AdamW optimizer to achieve both computational efficiency and precise detection capabilities. The integration of SE_Block further enhanced feature representation by adaptively recalibrating channel-wise attention, improving detection accuracy and robustness. The algorithm was labeled and trained by using a diverse dataset of 1500 tomato leaf images consisting of four labels (All, Green Tomato, Downy Mildew, and Powdery Mildew), capturing variations in disease types, lighting conditions, and leaf orientations, enabling robust detection performance across real-world scenarios. The incorporation of Depthwise Grouped Convolutions into YOLOv8 reduced the computational complexity, enabling faster inference speed without sacrificing detection accuracy. Additionally, the AdamW optimizer enhanced the model convergence during training, ensuring robustness and stability. Compared with the original algorithm, the improved YOLOv8 achieved a significant performance improvement, with model precision (P%) increasing from 83.5% to 85.7% (2.2% increase), recall (R%) improving from 70.4% to 72.8% (2.4% increase), and mAP@0.5 improving from 75.7% to 79.8% (4.1% increase). mAP@0.5:0.95 also saw an improvement, rising from 44.2% to 51.6% (7.4% increase). Furthermore, the F1 score increased from 76.4% to 78.6% (2.2% increase), demonstrating enhanced overall detection accuracy. The system was deployed on the Spraying Robot LPE-260 to enable real-time, automated pesticide application in controlled environments. The improved detection framework ensures the targeted spraying of diseased tomato leaves, significantly reducing chemical usage and minimizing overspray. This system ensures that pesticide is sprayed exclusively on the diseased areas of tomato leaves, further minimizing chemical usage and overspray. It demonstrates the potential of computationally efficient deep learning techniques to address key challenges in precision agriculture, advancing scalable, sustainable, and resource-efficient crop management solutions.

## 1. Introduction

Deep learning algorithms have transformed precision agriculture, showing great potential in crop management, pest control, and orchard monitoring. These techniques enable the real-time detection and classification of agricultural challenges. By addressing spatial variability, environmental factors, and disease detection with precision, deep learning enhances efficiency in agriculture. Object detection frameworks like YOLO, known for speed and accuracy, are driving this shift. Advances such as Grouped Depthwise Convolutions and feature recalibration through SE_Block are boosting detection accuracy and optimizing resource use in precision agriculture.

In the 21st century, the effective implementation of precision agriculture relies on integrating tools like Geographic Information Systems (GISs), Global Positioning Systems (GPSs), and variable rate application systems to address spatial variability and improve efficiency [1]. These technologies form the backbone of precision agriculture, enabling site-specific crop management and enhancing productivity and sustainability [2]. Advanced systems, such as the enhanced YOLOv4-Tiny model, improve the multi-class detection of cherry tomatoes, allowing for the precise recognition of ripeness stages: ripe, semi-ripe, and unripe, within complex agricultural environments [3]. Additionally, a study developed seedling-YOLO with the upgraded YOLOv7-Tiny model, incorporating ELAN_P, CARAFE, and coordinate attention modules, boosting mAP [4]. Disease prediction models, remote sensing, and site-specific strategies highlight the transformative impact of precision agriculture technologies on plant disease management [5]. AI-driven solutions have significantly advanced fruit detection and yield estimation in agriculture. Convolutional neural networks (CNNs) improve tomato detection and mass estimation, offering accurate weight predictions based on tomato dimensions [6]. Transfer learning, by fine-tuning pre-trained deep learning models like DenseNet121 with synthetic images from C-GAN, enhances tomato leaf disease detection, ensuring high accuracy in precision farming [7]. Deep learning techniques, including CNNs, also aid in the classification of various tomato diseases, addressing the complexities of disease recognition [8]. Machine vision systems automate the recognition, detection, and classification of weeds in cotton fields, supporting sustainable weed management practices [9]. Comparative studies of SSD and YOLO models show that SSD MobileNet v2 achieves a better balance between detection accuracy and inference time in tomato detection in greenhouse environments, while YOLOv4-Tiny offers faster inference speed [10]. Enhancements to the YOLO-X model in disease severity detection further address challenges like background interference and overlapping leaves [11]. The introduction of Tomato-YOLOv7 improves the real-time detection of ripe tomatoes, tackling occlusion and lighting variations while reducing manual labor [12]. Additionally, improvements to the YOLOv3 model enhance tomato disease and pest detection, overcoming issues like small object sizes and overlapping leaves for real-time monitoring [13].

Advanced deep learning techniques, such as deep migration learning, play a crucial role in enhancing precision agriculture by enabling the detection of key tomato organs, even in complex agricultural environments [14]. An improved YOLOv4 model, integrated with EfficientNet-B0 and PANet, achieved 93.42% precision and 63.20 FPS, significantly boosting apple detection in complex environments [15]. The YOLOX-tiny model, enhanced with ShuffleNetV2, CBAM, and ASFF, reached 96.76% precision, optimizing robotic apple-picking systems [16]. A modified YOLOv8 introduced dilated convolutions and an adapted gradient algorithm, targeting crown regions in natural environments [17]. Depthwise separable convolution-based models have improved efficiency and accuracy in plant leaf disease detection by effectively capturing disease-specific features while reducing computational complexity [18]. MixConv, which integrates mixed depthwise convolutional kernels, dynamically adjusts kernel sizes to improve feature extraction and performance in CNNs [19]. In resource-constrained environments like IoT devices, efficient deep learning methods, such as those for plant disease classification, optimize model execution and address deployment challenges in precision agriculture [20]. An efficient UNet-based algorithm, akin to InstaCropNet, has demonstrated high accuracy in crop row detection across diverse field conditions, improving precision in agricultural applications [21]. Advances in machine learning, such as evolved optimizers, adapt to various vision tasks in precision agriculture, enhancing both training efficiency and accuracy [22]. Deep learning techniques, including those for face mask detection, show adaptability in dynamic safety applications, extending to agricultural environments [23]. Optimized predictive modeling techniques, such as adaptive inference systems, improve convergence and stability in agricultural decision making, supporting real-time applications in complex environments [24]. Attention mechanisms, like dynamic spatial–channel attention networks, effectively recalibrate channel-wise feature responses for better accuracy and efficiency in precision agriculture. These architectures are critical to crop monitoring and decision making, as shown in sugarcane field segmentation using satellite imagery [25]. Refinements of fully convolutional networks (FCNs) with spatial and channel Squeeze-and-Excitation (SE) Blocks have enhanced feature recalibration by dynamically adjusting spatial and channel feature importance. This approach has proven effective in plant disease detection, such as rice leaf disease classification, where precise feature enhancement is essential to accurate analysis [26].

Precision agriculture faces several challenges that hinder its widespread adoption. Data scarcity and the limited generalization of existing models reduce their adaptability to diverse agricultural conditions, while high computational demands constrain the deployment of advanced algorithms in resource-limited environments. Moreover, the interpretability of deep learning models remains an obstacle, complicating their integration into decision-making processes. To address these challenges, this study introduces a novel robotic spraying system for tomato crops, incorporating an enhanced YOLOv8 framework. This system improves computational efficiency and accuracy by integrating Grouped Depthwise Convolutions (GDCs) and Squeeze-and-Excitation (SE) Blocks, reducing computational complexity and boosting model performance, particularly under dynamic agricultural conditions. Additionally, the AdamW optimizer is used to ensure better convergence and stability during model training.

**Figure 1:**
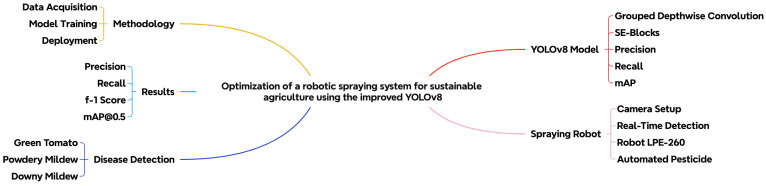
Integrating YOLOv8 for disease detection in automated agricultural spraying.

Figure 1 provides a visual summary of the key components and workflow of this research study. It highlights the main elements of the study, including the improved YOLOv8 model, the robotic spraying system, disease detection, and the methodology used for data acquisition, model training, and deployment. This overview helps readers understand the structure and objectives of this research study.

This study focuses on real-time automated disease detection for Powdery Mildew, Downy Mildew, and Green Tomato using the improved YOLOv8 model. By deploying the model on the Spraying Robot LPE-260, we aim to minimize pesticide use while maximizing crop management efficiency. This framework presents a novel approach to precision agriculture by addressing the dual challenges of computational efficiency and high detection accuracy, offering a more scalable and practical solution for sustainable crop management. The following objectives are pursued in this study:

1. Develop an improved YOLOv8 model incorporating Grouped Depthwise Convolutions and Squeeze-and-Excitation (SE) Blocks to enhance accuracy and computational efficiency in tomato leaf disease detection.
2. Integrate the optimized YOLOv8 model into a robotic spraying system for real-time, automated detection and pesticide application.
3. Evaluate the performance of the proposed model in terms of precision, recall, F1 score, and mean average precision (mAP) against existing models.
4. Assess the system’s practical feasibility in reducing chemical usage and improving resource efficiency in sustainable agricultural practices.

The manuscript is organized as follows: Section 2 covers data acquisition, describing the hardware framework, spraying mechanism, camera setup, data collection process, and classification methods. Section 3 presents the methodology, including an overview of the standard YOLOv8 architecture, the design and workflow of the improved YOLOv8, and the integration of Grouped Depthwise Convolutions, Squeeze-and-Excitation (SE) Blocks, and the AdamW optimizer. Section 4 discusses model training and variable configuration, detailing the research setup, model training process, performance metrics, computational evaluation, and loss calculations. Section 5 presents the results and discussion, offering a comparative analysis of decomposition experiments and detection performance across different models. Finally, Section 6 concludes with the conclusion and future work, summarizing the study’s contributions and suggesting directions for future research.

## 2. Data Acquisition

### 2.1. System Architecture and Operational Components

**Figure 2:**
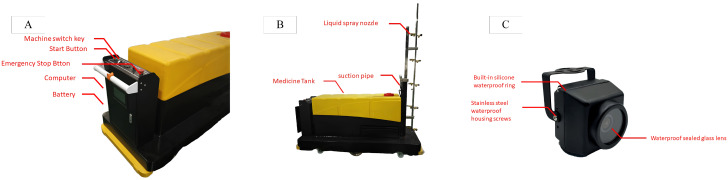
Hardware architecture of autonomous spraying robot LPE-260 (**A**) Spraying robot interface, (**B**) Sprayiing robot key components, (**C**) Spraying robot camera integration.

Figure 2A illustrates the essential components of the spraying robot interface, which includes the Machine Switch Key, Start Button, and Emergency Stop Button. The Machine Switch Key allows authorized users to activate or deactivate the machine, while the Start Button initiates its operation. The Emergency Stop Button is a critical safety feature, enabling immediate shutdown during emergencies to prevent accidents or equipment damage. Beneath these controls, the computer serves as the central processing unit, managing operations and ensuring the smooth execution of programmed tasks. Powered by a Battery, the system remains portable and can operate in areas without direct access to a power source. This compact and ergonomic design enhances user interaction, contributing to overall efficiency and ease of use. Figure 2B highlights the primary functional components of the robot, including the Liquid Storage Tank, Suction Pipe, and Liquid Spray Nozzles. The yellow Liquid Storage Tank serves as the main reservoir for the liquid to be sprayed, with the Suction Pipe transferring it to the Nozzles, which are mounted on a vertical metal frame to ensure even and precise distribution. This design optimizes the robot’s performance, making it ideal for tasks such as crop spraying, disinfection, and industrial applications. Additionally, the Spraying Robot LPE-260, shown in Figure 2C, is equipped with an integrated camera system that enhances its operational capabilities. The equipment was sourced from Jiangsu Greenport Modern Agricultural Development Co., Ltd., Suqian, China. This camera provides real-time visual feedback for monitoring, navigation, and data collection, enabling remote oversight of the spraying process. The captured data support autonomous navigation, helping the robot adapt to environmental conditions, and can be used for post-operation assessments, improving overall efficiency and effectiveness in agricultural and industrial tasks.

### 2.2. Hardware Limitations and Mitigation Strategies for Spraying Robots

A significant challenge in deploying spraying robots is the dynamic control problem associated with the vehicle’s weight distribution, particularly due to the pesticide tank. The shifting mass within the tank leads to continuous variations in the robot’s center of gravity during operation. As the liquid is used or redistributed, these changes result in destabilizing forces that induce oscillations or the shaking of the vehicle. This dynamic instability complicates the control of the robot, particularly during high-speed maneuvers or when navigating uneven terrain. Such instability can negatively impact the precision of spraying, as well as the overall safety and performance of the system. Solutions to this challenge include designing adaptive control systems that can compensate for shifting weight distribution and stabilizing mechanisms that dampen the effects of oscillations.

Furthermore, prolonged operation of the spraying robot exposes critical components such as cameras and sensors to pesticide-induced corrosion, which accelerates component degradation and compromises detection accuracy over time. To mitigate these issues, the integration of corrosion-resistant materials, protective coatings, and advanced sealing technologies is essential to enhancing the durability and longevity of these components, ensuring reliable performance over extended periods.

In addition, communication stability within greenhouse environments is often disrupted by interference from metal structures and environmental factors, leading to instability in data transmission. To ensure consistent and reliable connectivity, the deployment of edge computing systems is recommended. By processing data locally, edge computing reduces dependence on remote communication networks, thus improving overall system robustness and operational reliability, particularly in environments with limited or fluctuating network coverage.

### 2.3. Intelligent Spraying System and Optimized Pesticide Spraying Process for Disease Detection

**Figure 3:**
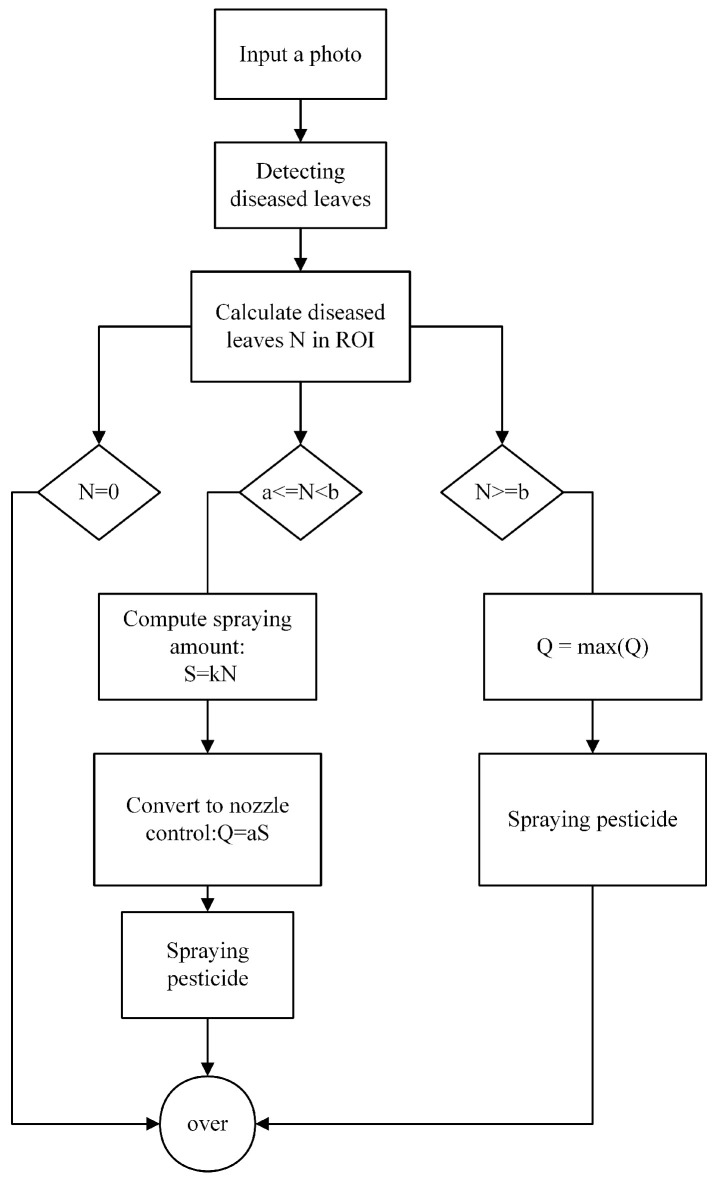
Automated disease detection and pesticide application control system.

Figure 3 outlines a process for detecting diseased leaves in a photo, calculating the required amount of pesticide to be sprayed, and controlling the nozzle to apply the pesticide efficiently. The process begins with the input of an image, followed by detecting diseased leaves within the image. The number of diseased leaves, denoted by *N*, is calculated within the Region of Interest (ROI). If no diseased leaves are detected (N=0), the process ends. If *N* is between predefined thresholds *a* and *b*, the spraying amount *S* is computed by using the relation S=kN. This amount is then converted to nozzle control parameters, where Q=aS, and the pesticide is sprayed. If *N* exceeds the threshold *b*, the maximum spraying quantity, *Q*, is adjusted by taking the maximum value of *Q*, ensuring that the spraying amount does not exceed capacity for effective application.

### 2.4. Data Collection and Classification

**Figure 4:**
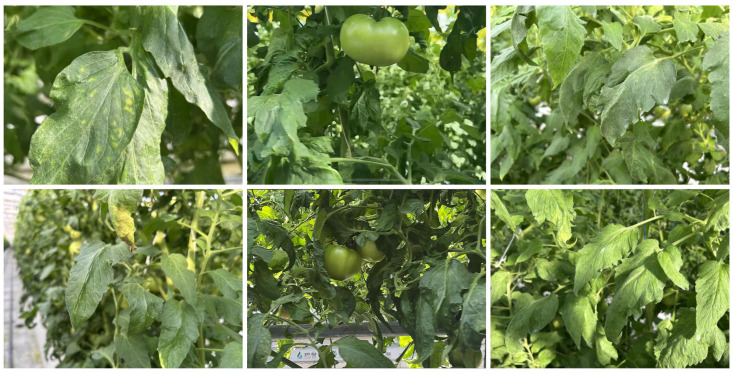
Sample images from dataset in tomato farm.

The dataset used in this study consists of 1500 images captured in Suqian, Jiangsu, China, at Jiangsu Green Harbor Modern Agricultural Development Co., Ltd. The images were collected under various environmental conditions, including varying lighting, occlusion, and natural interference, as shown in Figure 4. The dataset includes four class labels: ‘Green Tomato’, ‘Downy Mildew’, ‘Powdery Mildew’, and ‘All’. The ‘All’ label represents a comprehensive class for evaluating the performance of all other labels combined. The primary focus of the spraying system is on the diseased areas, specifically targeting the ‘Downy Mildew’ and ‘Powdery Mildew’ classes, which represent the unhealthy parts of the leaves requiring pesticide application. By concentrating on these affected areas, the system ensures more efficient use of resources, minimizing pesticide waste and reducing the environmental impact while effectively controlling the diseases.

**Figure 5:**
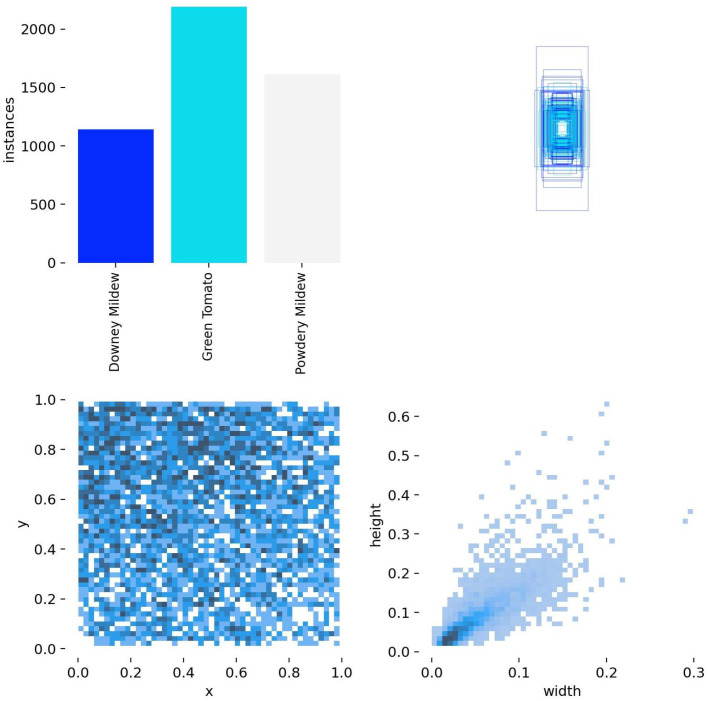
Distribution and feature correlation analysis of tomato disease instances.

Figure 5 presents a detailed visualization of the distribution of instances from three categories related to tomato diseases: Downy Mildew, Green Tomato, and Powdery Mildew. The top-left bar chart highlights the number of instances for each category, with Green Tomato having the highest count, followed by Downy Mildew and Powdery Mildew. The scatter plots below provide further insights into the spatial distribution and relationships between different features of the instances. The plot on the bottom left shows the distribution of the y (vertical) and x (horizontal) coordinates for the instances, with a dense concentration in the middle, indicating that most instances are centrally located. The plot on the bottom right visualizes the correlation between the height and width of the instances, showing a positive trend where taller instances tend to also be wider. Figure 5 offers a statistical representation of the dataset, emphasizing the density of instances in certain regions and the relationships between the key features of the tomato disease instances.

**Figure 6:**
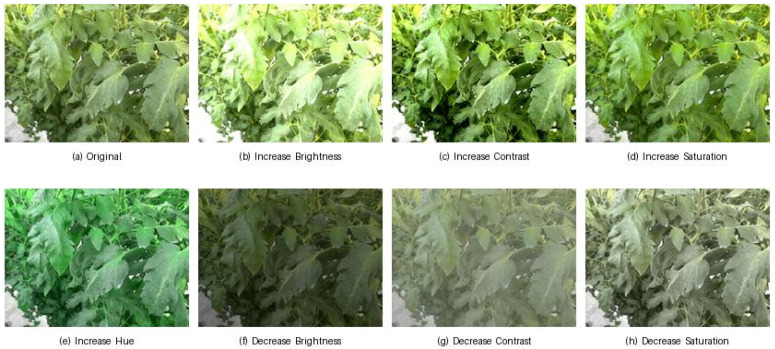
Augmented variants of dataset images using brightness, contrast, hue, and saturation adjustments.

The images were annotated by using LabelMe v5.6.1 software, a tool that enables bounding box annotations for object detection. This process involved precise labeling to ensure accurate detection and classification during model training. After annotation, the dataset was converted into the YOLOv8-compatible format for efficient training and evaluation. To improve the robustness of the dataset, various data augmentation techniques were applied. These included adjustments to brightness, contrast, hue, and saturation, as shown in Figure 6. The data collection and augmentation process created a diverse and representative dataset for agricultural disease detection and monitoring. Table 1 outlines the dataset details, which includes 1500 images in total. Of these, 1180 images are used for training and 320 for validation. Preprocessing involves auto-orienting the images, and data augmentation techniques such as brightness, contrast, saturation adjustments, and hue variations are applied. Each training image contains 3 object classes.

In the practical implementation of the system, data collection plays a pivotal role in ensuring that the robot can correctly identify and target diseased areas for pesticide application. The Spraying Robot LPE-260 is equipped with a high-resolution camera system integrated onto the robot. This camera captures images of the tomato leaves in real time, with variations in environmental conditions (lighting, occlusion, etc.), which are necessary for training and validating the detection model in different agricultural scenarios. Real-time image capture: The camera captures images under various lighting and environmental conditions, which is crucial for the model to be robust to diverse real-world scenarios. This includes images of green tomatoes and diseased leaves with annotations for Downy Mildew, Powdery Mildew, and Green Tomato. Data annotation: The captured images are annotated with bounding boxes that define the Regions of Interest (ROIs), marking diseased areas (Downy Mildew and Powdery Mildew) and healthy areas (Green Tomato). These annotations serve as ground-truth data, crucial to the training of the YOLOv8 model. The dataset, which contains around 1500 labeled images, is diverse in terms of leaf orientations, disease severity, and light conditions (Figure 4). Augmentation: To improve the generalization of the model, various augmentation techniques, such as adjusting brightness, contrast, hue, and saturation, are applied to the dataset. This helps to simulate different environmental conditions and further enhances the model’s ability to handle real-world variability (Figure 6).

## 3. Methodology

### 3.1. Standard YOLOv8 Architecture and Functionality

This study employs the YOLOv8 model for its lightweight architecture and exceptional real-time efficiency. The system consists of Backbone, Neck, and head, forming a unified pipeline for feature extraction, fusion, and prediction. The Backbone performs multi-scale feature extraction through convolution operations, capturing both spatial and contextual information from input images. The Neck uses feature pyramid structures to enhance the fusion of high-level semantic features with detailed low-level representations, improving detection precision. The Head incorporates anchor-free detection strategies, enabling bounding box and class probability predictions with minimal computational overhead. The modular design also includes CBS modules (Convolution, Batch Normalization, and SiLU activation) and C2f structures, optimizing feature reuse while balancing speed and accuracy. This combination ensures robust performance across diverse scenarios with high processing efficiency.

### 3.2. Improved YOLOv8 Design and Operational Workflow

**Figure 7:**
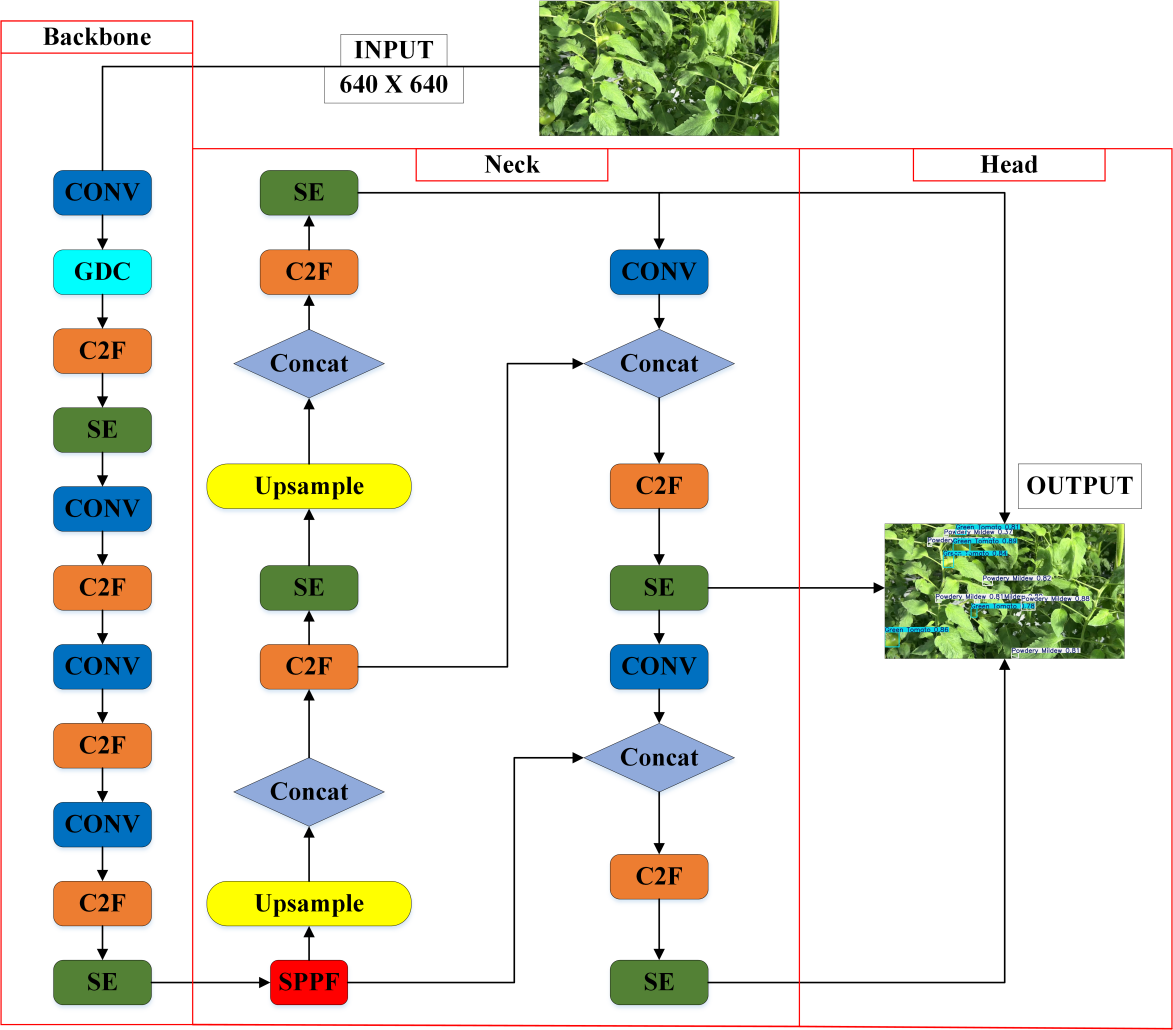
Improved YOLOv8 model pipeline for feature extraction and detection.

Figure 7 shows the architecture of an enhanced object detection model with three main components: Backbone, Neck, and Head. The input image (640 × 640) first passes through the Backbone for feature extraction. This stage includes convolutional layers, Grouped Depthwise Convolutions (GDCs), Cross Stage Partial with Focus (C2F), and Squeeze-and-Excitation (SE) Blocks to optimize feature representation by reducing redundancy and highlighting key features. The Neck aggregates multi-scale features for improved detection across various object sizes, using SE_Block, C2F layers, concatenation, and upsampling for effective feature fusion. A Spatial Pyramid Pooling-Fast (SPPF) block further improves multi-scale aggregation, creating a unified feature map for more accurate detection. The Head processes these features to generate final predictions, including class labels and bounding box coordinates, with additional convolutional layers and SE modules refining accuracy. GDC and SE_Block enhance model efficiency by capturing both fine details and long-range dependencies, which is crucial to detecting occlusions or subtle infections like Downy and Powdery Mildew. The model is optimized for real-time inference on edge devices, using depthwise separable convolutions to reduce computational cost and enable rapid decision making, essential to robotic systems in dynamic environments.

### 3.3. Grouped Depthwise Convolution

**Figure 8:**
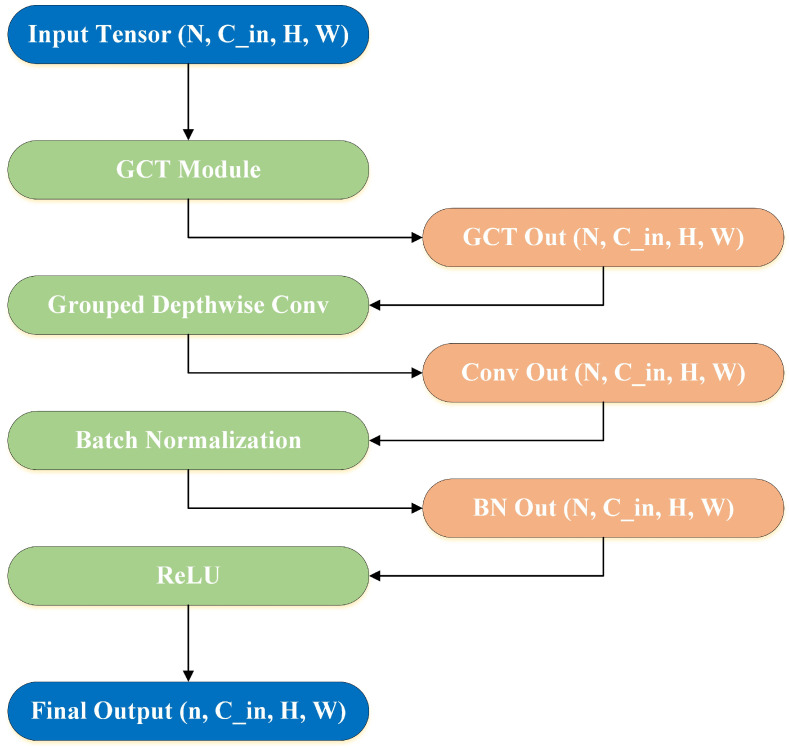
Architectural framework incorporating Grouped Depthwise Convolutions for feature processing.

Figure 8 illustrates a modular data processing pipeline, starting with an input tensor of dimensions $\left(N,C_{in},H,W\right)$, where N represents the batch size, $\left(C_{in}\right)$ is the number of input channels, and H and W are the height and width of the tensor, respectively. The input tensor first passes through the Generalized Convolutional Transformation (GCT) module, which performs channel-wise transformations to enhance the model’s representational capacity. The output of this module retains the same dimensions as the input tensor. Next, the tensor undergoes a Grouped Depthwise Convolution operation, which applies depthwise convolutions within channel groups, focusing on spatial feature extraction while preserving the tensor’s overall shape. This results in an output tensor denoted by Conv Out $\left(N,C_{in},H,W\right)$. The tensor then undergoes Batch Normalization (BN), which standardizes features across the batch to stabilize and accelerate training. This step adjusts the mean and variance of the tensor’s features, resulting in an output tensor BN Out $\left(N,C_{in},H,W\right)$ with consistent dimensions. The normalized tensor is then passed through a Rectified Linear Unit (ReLU) activation function, introducing non-linearity into the model by setting negative values to zero and keeping positive values unchanged. This ensures the tensor’s shape remains unchanged throughout the process. The final output tensor, with dimensions $\left(n,C_{in},H,W\right)$, is the result of this carefully structured sequence of operations, highlighting the combined effects of feature transformation, spatial filtering, normalization, and activation. In precision agriculture, where objects of interest—such as diseased areas on tomato leaves—are often small, occluded, or subject to variations in lighting and orientation, Grouped Depthwise Convolutions improve the model’s ability to efficiently detect these features. Unlike traditional convolutions, which apply filters across all channels, depthwise convolutions focus on spatial feature extraction for each channel group, reducing redundancy and enhancing performance in resource-constrained environments.

### 3.4. SE_Block: Channel-Wise Attention Mechanism in Improved YOLOv8

**Figure 9:**
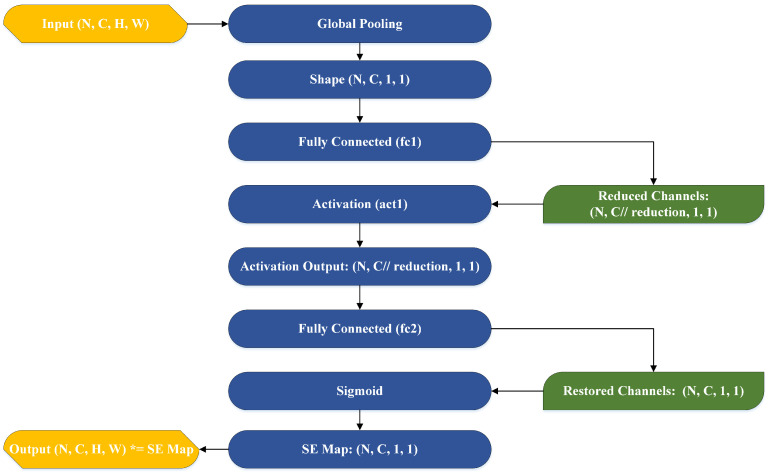
Squeeze-and-Excitation (SE) Block architecture for channel-wise attention mechanism.

Figure 9 illustrates the design of a Squeeze-and-Excitation (SE) Block, a channel-wise attention mechanism that enhances representational capacity in the improved YOLOv8 model. The SE_Block begins by taking an input tensor of dimensions (N,C,H,W), where N is the batch size, C is the number of channels, and H and W are the spatial height and width, respectively. It starts with a Global Pooling operation that compresses the spatial dimensions, resulting in a tensor of shape (N,C,1,1), thereby capturing global contextual information across the spatial domain. Next, the reduced tensor passes through a Fully Connected (fc1) layer, which performs channel reduction to $(\frac{N,C}{reduction}$,1,1), where the reduction factor is a hyperparameter that controls the degree of compression. A ReLU activation function (act1) is applied to introduce non-linearity. The output of this activation is then passed through another Fully Connected (fc2) layer to restore the original channel dimensions (N,C,1,1). Following this, a Sigmoid activation function is applied to compute channel-wise weights, generating the final SE map of shape (N,C,1,1), which represents the attention scores for each channel. The SE map is then applied element-wise to the original input tensor via multiplication, enhancing important channels while suppressing less significant ones. The final output retains the same shape as the input (N,C,H,W) but incorporates refined channel-wise attention. SE_Block enhance the model’s ability to distinguish relevant disease-specific features from background noise. In agriculture, differentiating subtle disease markers (like Downy Mildew or Powdery Mildew) from environmental factors (such as lighting or leaf orientation) is crucial. The SE_Block allows the model to focus on these key areas, improving detection accuracy and enabling more efficient pesticide application.

Table 2 compares the performance metrics of different YOLOv8-based models optimized by using the AdamW optimizer, with a focus on the impact of various architectural modifications. The baseline model, YOLOv8+AdamW, achieves a mean average precision (mAP@0.5) of 75.7%, with a precision of 81.2%, a recall of 70.9%, and an F1 score of 75.7. When a Grouped Depthwise Convolution is added (YOLOv8+GDC+AdamW), the model’s mAP@0.5 increases to 79.1%, accompanied by slight improvements in recall and precision, reaching 71.3% and 85.5%, respectively. On the other hand, integrating a Squeeze-and-Excitation (SE) Block into the model (YOLOv8+SE_Block+AdamW) leads to a decrease in performance, with mAP@0.5 dropping to 74.3% and a lower F1 score of 73.73. However, when both GDCs and SE_Block are combined (YOLOv8+GDC+SE_Block+AdamW), the model shows the best performance, achieving the highest mAP@0.5 of 79.8%, a precision of 85.7%, a recall of 72.8%, and an F1 score of 78.62%. This combination offers balanced performance across all metrics, with a slightly increased GFLOP (75.6) and a modest increase in parameters (4.2 M), making it the most effective configuration in terms of accuracy and efficiency. This comparison highlights the benefits of adding specific architectural components like GDCs and SE_Block to improve the model’s overall performance.

### 3.5. Parameter Updates with AdamW Optimizer

**Figure 10:**
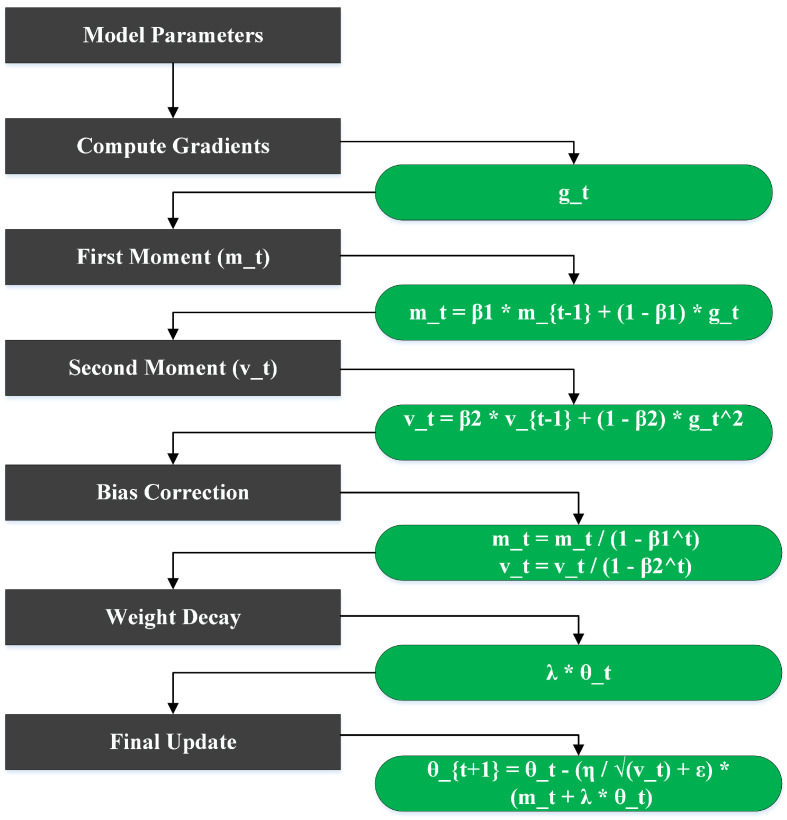
AdamW optimization algorithm workflow for parameter updates.

Figure 10 represents the workflow of the AdamW (Adam with Weight Decay) optimization algorithm, which enhances the standard Adam optimizer by decoupling weight decay from gradient updates for improved generalization in deep learning models. The process begins with the model parameters, which undergo gradient computation based on the loss function, yielding the gradient $g_{t}$ at time step *t*. The next step involves calculating the first moment estimate $m_{t}$ and the second moment estimate $v_{t}$. The first moment $m_{t}$ accumulates the exponentially weighted average of past gradients by using Equation (1):

(1) $$m_{t}=\beta_{1}\times m_{t-1}+\left(1-\beta_{1}\right)\times g_{t}$$

where $\beta_{1}$ is the smoothing parameter controlling the decay rate. Similarly, the second moment tracks the exponentially weighted average of squared gradients by using Equation (2):

(2) $$v_{t}=\beta_{2}\times v_{t-1}+\left(1-\beta_{2}\right)\times g_{t}^{2}$$

where $\beta_{2}$ determines the decay rate for the squared gradients. Following these calculations, bias correction is applied to the first and second moments to compensate for their initialization bias in the early steps. The corrected estimates are given by Equation (3):

(3) $$\hat{m_{t}}=\frac{m_{t}}{1-\beta_{1}^{t}},\hat{v_{t}}=\frac{v_{t}}{1-\beta_{2}^{t}}$$

After bias correction, a weight decay term is incorporated, computed as $\lambda \times \theta_{t}$, where λ is the weight decay coefficient and $\theta_{t}$ represents the current parameters. This step penalizes large weights to improve generalization and reduce overfitting. Finally, parameter update is performed by using Equation (4):

(4) $$\theta_{t+1}=\theta_{t}-\eta \times \left(\frac{\hat{m_{t}}}{\sqrt{v_{t}}+\epsilon }+\lambda +\theta_{t}\right)$$

where η is the learning rate and ϵ is a small constant to prevent division by zero. This update integrates gradient scaling (via the first and second moments) and weight decay into the optimization process. The AdamW optimizer is particularly suited for handling the variability in agricultural data. By decoupling weight decay from gradient updates, it helps prevent overfitting to noisy, sparse data often found in real-world agricultural environments. This ensures stable and fast convergence, which is vital to training models on diverse agricultural datasets. AdamW’s adaptive learning rate also aids in handling the challenges posed by highly variable environmental conditions, such as changing weather or plant growth stages.

## 4. Model Training and Variable Configuration

### 4.1. Research Setup and Model Training

The experiments were conducted on the Ubuntu operating system, with deep learning models implemented by using the PyTorch v2.0.1 framework. Table 3 provides the full configuration details of the research setup. The training process used the AdamW optimizer, which efficiently handles large-scale models and balances optimization and generalization by decoupling weight decay from gradient updates. The learning rate parameters were configured with an initial learning and final learning rate $lr_{0}$ = $lr_{f}$ = 0.01, ensuring stability and compatibility throughout the optimization process. Other hyperparameters used in the training process included the momentum, set to 0.937; a weight decay of 0.0005; and a mask ratio of 4. Input images were resized uniformly to 640 × 640 dimensions (imgsz=640) to maintain consistency across the training data. A batch size of 16 was utilized, and training ran for a total of 1000 iterations. As shown in Figure 7, the training pipeline begins with images being fed into the neural network, where the backbone module extracts key features for downstream processing. After feature extraction, the AdamW optimizer updates the model parameters. This optimizer extends Adam by introducing weight decay into the parameter update step, which improves generalization and prevents large weights, thus mitigating overfitting. AdamW also uses adaptive learning rates, leveraging the first and second moment estimates of gradients to ensure efficient convergence in complex optimization landscapes. The refined features from the backbone are then passed to the detection head to generate the final predictions.

To assess the model’s suitability for deployment in resource-constrained environments, the experiment utilized RK3588, a high-performance computing platform designed for edge applications. RK3588 features an 8-core ARM Cortex-A76 and Cortex-A55 CPU, a Mali-G610 GPU, and an independent NPU (neural network processing unit), providing the efficient computing and real-time processing capabilities needed for robotics and precision agriculture. For model deployment, we used the NPU, which offers lower power consumption than the GPU while meeting real-time detection requirements. Its compact size (94 mm × 68 mm × 31 mm) and low-power design (with typical consumption ranging from 10 W to 30 W) make it ideal for space- and power-constrained environments. This setup further validates the model’s performance and practicality in scenarios where energy efficiency and a compact form factor are essential.

### 4.2. Performance Metrics and Computational Evaluation

The model’s performance was evaluated by using several key metrics: precision, recall, mAP, GFLOPS, inference speed, F1 score, and the total number of parameters. Precision measures the ratio of correctly identified positive predictions to the total number of predictions (both positive and negative), indicating the algorithm’s accuracy in detecting relevant instances. Recall quantifies the proportion of true-positive cases identified, reflecting the model’s ability to capture all relevant instances. Mean average precision (mAP) provides a comprehensive evaluation by calculating the mean precision across all classes, focusing on the overlap between predicted and ground-truth boxes. GFLOPS (Giga Floating Point Operations Per Second) assesses the computational complexity of the model, shedding light on its resource efficiency. Inference speed was measured to determine how quickly the model processes data, which is critical to real-time applications. The F1 score, the harmonic mean of precision and recall, was included to ensure a balanced evaluation, especially in cases where precision and recall are not perfectly aligned. Finally, the total number of parameters was analyzed to evaluate the model’s size and memory requirements. Together, these metrics offer a comprehensive view of the model’s accuracy, efficiency, and feasibility for practical implementation.

(5) $$P=\frac{True\;Positives\;(TP)}{True\;Positives\;(TP)+False\;Positive\;(FP)}$$

(6) $$R=\frac{True\;Positives\;(TP)}{True\;Positives\;(TP)+False\;Negatives\;(FN)}$$

(7) $$AP_{i}=\int_{0}^{1}P_{i}\left(R\right)\;dR$$

(8) $$mAP=\frac{1}{N}\sum_{i=1}^{N}AP_{i}$$

(9) $$GFLOPS=\frac{Total\;Floating\;Point\;Operations}{10^{9}}$$

(10) $$Inference\;Speed=\frac{Total\;Inference\;Time}{Number\;of\;Images}$$

(11) $$F1=2\times \frac{P\times R}{P+R}$$

Equation (5) shows precision, which measures the proportion of correctly predicted positive samples out of all predicted positive samples, while Equation (6) shows recall, which measures the proportion of actual positive samples correctly identified. Equation (7) showcases average precision, where $P_{i}$ is the precision–recall curve for class I. Equation (8) showcases mean average precision, where N represents the number of classes. Equation (9) shows GFLOPS, which measures the computational cost of the model. Equation (10) shows inference speed, which measures the time taken to process a single input image, reported in milliseconds (ms) per image. Equation (11) shows the F1 score, which is the harmonic mean of precision and recall, balancing both metrics.

### 4.3. Loss Calculation

**Figure 11:**
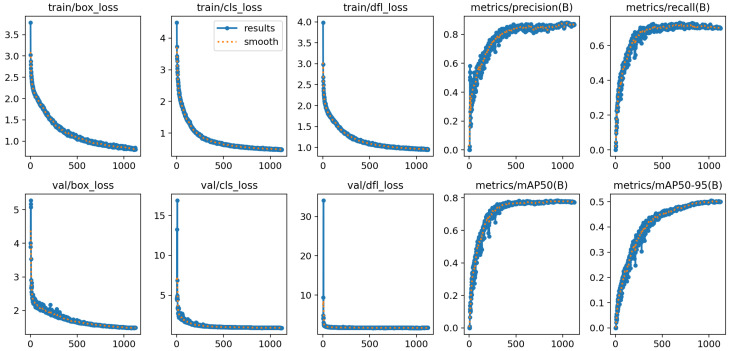
Training and validation performance metrics and loss curves for the improved YOLOv8 model over 1000 iterations. The X-axis represents the number of training iterations, while the Y-axis shows the corresponding values for different performance metrics (such as loss, precision, recall, and mAP).

Figure 11 illustrates the training and validation performance metrics and loss curves for the improved YOLOv8 model across 1000 iterations. The results demonstrate the model’s optimization process and its ability to generalize to unseen data during validation. The graphs provide an in-depth view of the model’s behavior, including training and validation losses, as well as evaluation metrics. The training loss curves in the top row show the bounding box regression loss (train/box_loss), which steadily declines, indicating improved alignment with the ground truth. The classification loss (train/cls_loss) is consistently reduced, reflecting the model’s improved ability to differentiate among object classes, and the distribution focal loss (train/dfl_loss) decreases, highlighting improved precision in bounding box predictions. The evaluation metrics for training reveal increasing precision (metrics/precision), signifying a reduction in false positives. Recall (metrics/recall) steadily rises, indicating better detection of true positives. Similarly, the validation loss curves in the bottom row complement the training losses, with the validation box loss (val/box_loss) following a similar decline, confirming the model’s ability to generalize to unseen data for object localization. The validation classification loss (val/cls_loss) and distribution focal loss (val/dfl_loss) also decrease, suggesting reliable class and bounding box predictions. The validation metrics further highlight the model’s performance, with a steady increase in mean average precision at 50% IoU (metrics/mAP@0.5), demonstrating improved detection accuracy across all classes. There is also a consistent upward trend in mean average precision at 50–95% IoU (metrics/mAP@0.5:0.95), indicating the model’s robustness in detecting objects at varying levels of overlap.

## 5. Results and Discussion

### 5.1. Comparative Analysis of Decomposition Experiment and Improved YOLOv8 Algorithm

The results presented in Table 4 show the performance of different models, including the combined model ‘AdamW+SE_Block+GDC’. This combined model achieves the highest mean average precision (mAP) of 79.8%, indicating superior accuracy in object detection compared with the individual models. It also demonstrates an F1 score of 78.62%, reflecting a strong balance between precision and recall, crucial to tasks that demand accuracy with minimal false positives and negatives. Although the precision of the combined model (85.7%) is slightly lower than that of the best-performing model (GDC, 88.3%), its recall (72.8%) is higher, leading to better overall performance in terms of the F1 score. However, this improvement comes with increased computational demands, as the combined model requires 75.6 GFLOPS and takes 9.8 ms for inference, both higher than those of simpler models like AdamW.

**Figure 12:**
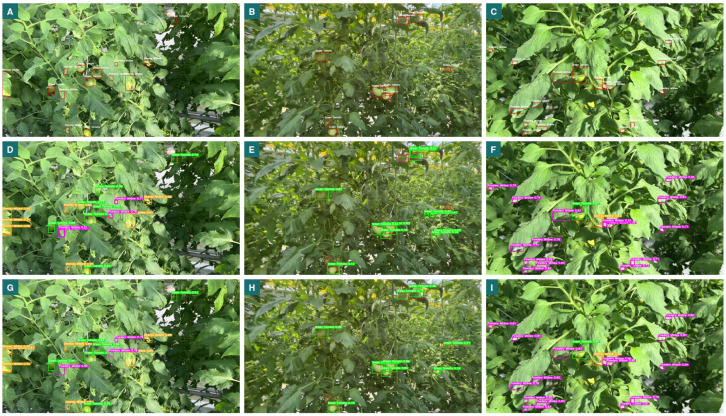
Object detection ground-truth images (**A**–**C**), original YOLOv8 detection result images (**D**–**F**), and improved YOLOv8 algorithm detection result images (**G**–**I**).

Figure 12 presents a comparative analysis of detection results from the original YOLOv8 model and the proposed improved YOLOv8 model across three image sets. The first row (A, B, C) shows the ground-truth annotations, including accurate bounding boxes and labels for the objects in the input images, which serve as the benchmark for evaluating model performance. The second row (D, E, F) displays detection results from the original YOLOv8 model, while the third row (G, H, I) shows outputs from the improved YOLOv8 model. A closer inspection reveals that the improved model consistently produces more accurate and comprehensive detection results than the original version. Notably, it shows better bounding box localization and fewer false positives. The improved model is also more effective in detecting smaller and more occluded objects, as seen in images G and H, where these objects would typically be missed. Additionally, the bounding box alignment in the improved YOLOv8 outputs is closer to the ground truth, indicating superior object localization and classification. This is particularly critical to our application, where the primary goal is to accurately detect and localize diseased areas on tomato leaves, such as Downy Mildew (yellow bounding boxes), Powdery Mildew (purple bounding boxes), and Green Tomato (green bounding boxes), for targeted pesticide application. The comparison highlights the effectiveness of the proposed improvements to YOLOv8, demonstrating significant advances in detection accuracy, object localization, and robustness in challenging scenarios.

**Figure 13:**
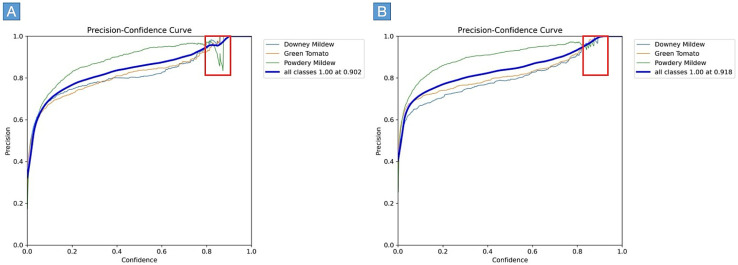
Precision–confidence curves: YOLOv8 (**A**) vs. improved YOLOv8 (**B**).

Figure 13 shows the precision–confidence curves for YOLOv8 (A) and the improved YOLOv8 (B), comparing their performance across three individual classes—Downy Mildew, Green Tomato, and Powdery Mildew—along with the overall performance across all classes. The thicker blue curve represents the aggregate precision for all classes, providing a general performance summary. For YOLOv8 (A), precision increases with higher confidence thresholds, but the model exhibits slightly lower precision for certain classes, particularly at moderate confidence levels. The ‘all classes’ curve reaches a maximum precision of 1.00 at a confidence threshold of 0.902, but there are noticeable gaps in precision consistency across the individual classes. This variability suggests that YOLOv8 struggles to maintain stable performance across different object types at specific confidence levels. In contrast, the improved YOLOv8 (B) shows superior performance, with higher precision across all confidence thresholds. The ‘all classes’ curve reaches a maximum precision of 1.00 at a confidence threshold of 0.918, indicating more consistent and robust detection compared with the original YOLOv8. Furthermore, the per-class curves (for Downy Mildew, Green Tomato, and Powdery Mildew) exhibit fewer fluctuations and higher precision, especially at lower confidence thresholds. This indicates that the improved YOLOv8 handles challenging detection scenarios more effectively, providing more accurate predictions across all object categories.

**Figure 14:**
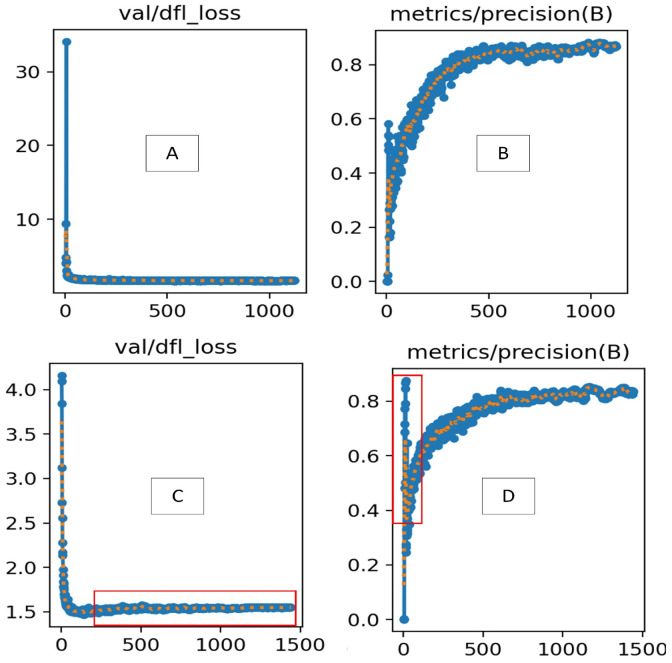
Comparison of validation loss and precision metrics between improved YOLOv8 (**A**,**B**) and YOLOv8 (**C**,**D**) algorithms (Blue represents the results, orange indicates the smoothness of the detected objects, and red highlights positions with greater fluctuations.

Figure 14 compares the improved YOLOv8 (A and B) with the standard YOLOv8 (C and D) in terms of validation loss (val/dfl_loss) and precision (metrics/precision). The top row shows results for the improved YOLOv8, while the bottom row displays the standard YOLOv8. In A (improved YOLOv8—validation loss), val/dfl_loss decreases rapidly during the initial training phase, stabilizing at a much lower value than the standard YOLOv8. This indicates the improved model’s better ability to refine bounding box predictions with higher precision and less variability, leading to faster convergence. In contrast, C (YOLOv8—validation loss) shows a higher loss plateau, indicating slower and less effective convergence. The red-marked region in C highlights a higher stabilized loss, suggesting that YOLOv8 struggles to optimize localization accuracy as effectively. In B (improved YOLOv8—precision), the precision steadily increases and reaches higher values, demonstrating improved detection accuracy. The curve is smoother, reflecting more consistent learning throughout training. In comparison, D (YOLOv8—precision) shows a similar upward trend but starts lower, with precision increasing more gradually. The red-marked region in D reveals greater fluctuations, indicating less stability in precision improvement. The improved YOLOv8 outperforms the standard YOLOv8 in both validation loss and precision. The reduced val/dfl_loss indicates better bounding box refinement, while the higher, more stable precision confirms enhanced detection capabilities.

### 5.2. Comparative Analysis of Detection Performance Utilizing Various Models

Table 5 presents a comparative analysis of various object detection models, evaluating key performance metrics such as precision, recall, mAP at the thresholds of 0.5 and 0.5:0.95, GFLOPs, inference time, and F1 score. The models compared include different versions of YOLO (v11, v9, v8, v7, and v5) and alternative architectures, such as RetinaNet and ATSS. The results indicate that the proposed method, ‘Ours’, outperforms all other models in terms of mAP@0.5 (79.8%) and mAP@0.5:0.95 (51.6%), while achieving a precision of 85.7% and a recall of 72.8%. These improvements are attributed to enhancements implemented in YOLOv8, particularly the integration of the AdamW optimizer, SE_Block, and GDC modules. Despite this performance gain, the inference time of ‘Ours’ remains competitive (9.8 ms), with GFLOPs (75.6) slightly higher than YOLOv9+AdamW+SE_Block+GDC but with significantly better detection performance. The F1 score of ‘Ours’ is the highest, at 78.6, further demonstrating the efficacy of the proposed modifications over existing models. Comparatively, YOLOv11, YOLOv9, YOLOv8, YOLOv7, and YOLOv5 exhibit lower performance, with YOLOv11+AdamW+SE_Block+GDC providing one of the best alternatives but still lagging behind the proposed method in mAP and F1 score. Overall, these results validate the effectiveness of the proposed improvements in enhancing both detection accuracy and efficiency, establishing ‘Ours’ as a superior model in this comparison. This demonstrates the effectiveness of the proposed enhancements to YOLOv8 for tomato disease detection tasks.

### 5.3. Performance Comparison of YOLOv8-Based Models for Tomato Disease Detection in Agricultural Robotics

Table 6 presents a comparative overview of studies that have employed YOLOv8-based models for tomato disease detection. It summarizes each study’s objectives, datasets, performance metrics, and the integration of models into robotic systems for real-time applications. The studies span a variety of approaches, from developing deep learning models for automated disease detection to enhancing accuracy through architectural improvements. Notably, our study distinguishes itself by integrating the YOLOv8 model directly into the Spraying Robot LPE-260, enabling real-time, automated pesticide application. This highlights the practical application of YOLOv8 in sustainable agriculture. In contrast, studies by [27,28] achieved high detection accuracy but did not explicitly discuss the integration of their models into robotic systems, suggesting room for future research to bridge the gap between model development and deployment in agricultural robotics. Overall, this analysis underscores the advancements in YOLOv8-based tomato disease detection and the ongoing efforts to incorporate these models into automated systems for real-time agricultural applications.

### 5.4. Limitations and Future Work

The improved YOLOv8 model shows substantial advancements in detection accuracy, but several limitations need to be addressed for broader applications in real-world agricultural environments. One limitation is its limited generalization to new disease types. The training dataset primarily includes Green Tomato, Downy Mildew, and Powdery Mildew, which may hinder the model’s ability to generalize to other diseases or pathogens not represented in the data. Expanding the dataset to cover a wider variety of diseases and environmental conditions would enhance the model’s robustness and adaptability. Another challenge is the model’s sensitivity to environmental variability, such as lighting changes, occlusions, and cluttered backgrounds. While the model was trained under diverse conditions, extreme weather, poor lighting, and cluttered environments could still pose challenges in real-world scenarios. Future work will focus on improving the model’s adaptability to these unpredictable conditions by diversifying the training samples and enhancing its robustness against environmental factors.

In terms of detection accuracy, while the model performs well overall, small-object localization, particularly for partially occluded or overlapping objects, remains a challenge. Despite applying Non-Maximum Suppression (NMS) to filter redundant bounding boxes, the model may still misdetect or fail to properly localize small or closely clustered objects. Future research will refine the localization process to improve small-object detection and ensure more precise bounding box placement.

While the current model is successfully deployed on the Spraying Robot LPE-260 for real-time pesticide application and its computational requirements are manageable, further optimization could enhance its inference speed and efficiency for faster real-time applications. Optimizing the model for a broader range of embedded edge devices, especially in scenarios with larger datasets or more computationally demanding environments, could improve its efficiency without compromising performance.

The lack of interpretability in deep learning models like YOLOv8 remains a challenge. Although the model achieves high detection accuracy, understanding the specific features and decision-making processes behind each prediction is difficult. Enhancing the model’s explainability will be crucial to practitioners in the field, enabling them to trust and better understand the model’s outputs. Future work will explore methods to improve transparency and interpretability, facilitating better integration into precision agriculture systems.

Data scarcity is a major challenge in precision agriculture for training generalized detection models. To enhance the YOLOv8 model’s performance, especially in generalizing to new diseases and varied environments, unsupervised and semi-supervised learning methods could prove valuable. Self-supervised learning can generate robust feature representations from unlabeled data, improving the model’s generalization capabilities. Clustering methods like k-means can group similar disease types, strengthening the model’s robustness to unannotated classes. Techniques like consistency regularization and pseudo-labeling can further improve performance by utilizing small labeled datasets and unlabeled data, respectively. Data augmentation using GANs can simulate diverse environmental conditions and enhance small-object detection. Finally, transfer learning enables fine tuning with larger labeled datasets, boosting the model’s ability to generalize. These strategies will enhance the YOLOv8 model’s adaptability and efficiency, making it more suitable for real-world agricultural applications.

## 6. Conclusions

This study introduces significant advancements in precision agriculture by developing an enhanced YOLOv8 framework for real-time disease detection in tomato crops. The model integrates Grouped Depthwise Convolutions, Squeeze-and-Excitation (SE) Blocks, and the AdamW optimizer, leading to substantial improvements in both detection accuracy (precision: 85.7%; recall: 72.8%; mAP: 79.8% at IoU@0.5) and computational efficiency. These innovations enable the model to effectively detect tomato diseases under various environmental conditions. The improved YOLOv8 model was successfully integrated into the Spraying Robot LPE-260, enabling real-time, automated, and targeted pesticide application. This integration reduces chemical usage, minimizes overspray, and ensures more efficient resource utilization, contributing to sustainable agricultural practices.

When compared with existing models, such as YOLOv5, YOLOv7, RetinaNet, and ATSS, the proposed framework consistently outperforms them in key metrics, including precision, recall, mAP@0.5, mAP@0.5:0.95, and F1 score. This demonstrates the model’s superior performance and robustness in practical agricultural settings. However, some limitations remain, such as the model’s inability to differentiate between specific pathogens or nutrient deficiencies as the underlying causes of disease. Expanding the dataset to include a broader range of diseases and environmental conditions would enhance the model’s generalizability.

Future research will focus on improving disease severity classification, developing an adaptive spraying mechanism for different crop types, and incorporating IoT-based remote monitoring to enhance scalability and real-time decision making. Overall, the proposed system offers a scalable and practical solution for efficient, sustainable crop management, combining deep learning techniques with automated robotic systems. This has the potential to revolutionize agricultural practices and contribute to more sustainable food production systems.

## Figures and Tables

**Table 1:** Dataset preprocessing and augmentation overview.

Attribute	Details
Total images	1500 images
Validation set	320 images
Training set	1180 images
Preprocessing applied	Auto-orientation (auto-rotate images to correct alignment)
Augmentation applied	Brightness: between −50% and +50%
	Contrast: between −30% and +30%
	Saturation: between −30% and +30%
	Hue: up to 6% of pixels
Outputs per training example	3 (indicating 3 object classes per image)

**Table 2:** Comparison of performance metrics for YOLOv8 variants with AdamW optimizer.

Network	P (%)	R (%)	mAP:@0.5 (%)	F1 Score	GFLOP	Inference Time (ms)	Params (M)
YOLOv8+AdamW	81.2	70.9	75.7	75.70	8.1	5.5	3.0
YOLOv8+GDC+AdamW	85.5	71.3	79.1	77.76	30.5	9.2	2.9
YOLOv8+SE_Block+AdamW	80.8	67.8	74.3	73.73	60.6	10.4	3.3
YOLOv8+GDC+SE_Block+AdamW	85.7	72.8	79.8	78.62	75.6	9.8	4.2

**Table 3:** System configuration and experimental environment.

Category	Configuration
CPU	CPU Intel(R) Xeon(R) Gold 6226R CPU @ 2.90 GHz
GPU	Nvidia 3090
System environment	Ubuntu 20.04.5 LTS
Framework	Pytorch 1.12
Programming	Python 3.8

**Table 4:** Impact of architectural improvements on YOLOv8 performance.

Network	P (%)	R (%)	mAP:@0.5 (%)	F1 Score	GFLOPs	Inference Time (ms)	Params (M)
YOLOv8	83.5	70.4	75.7	76.39	8.1	7.2	3.0
AdamW	81.2	70.9	75.7	75.70	8.1	5.5	3.0
SE_Block	86.3	71.3	78.8	78.09	60.6	10.4	3.3
GDC	88.3	70.8	79.6	78.50	30.5	9.2	2.9
AdamW+SE_Block+GDC	85.7	72.8	79.8	78.62	75.6	9.8	4.2

**Table 5:** Comparison of models.

Model	P (%)	R (%)	mAP@0.5 (%)	mAP@0.5:0.95 (%)	GFLOPs	Inference Time (ms)	F1 Score
YOLOv11	83.5	71.7	76.4	45.9	6.3	7.0	77.1
YOLOv11+AdamW+SE_Block+GDC	85.5	71.6	77.6	49.6	38.7	31.8	77.9
YOLOv9	83.8	72.5	78.2	50.5	27.1	8.0	77.7
YOLOv9+AdamW+SE_Block+GDC	84.7	72.9	78.4	46.4	59.7	19.2	78.3
YOLOv8	83.5	70.4	75.7	44.2	8.1	24.2	76.4
YOLOv7	44.9	35.8	33.4	12.2	103.2	2.7	39.8
YOLOv7+AdamW+SE_Block+GDC	65.8	54.3	61.7	29.9	59.3	13.5	59.5
YOLOv5	81.2	71.4	76.4	44.9	7.1	8.2	75.9
YOLOv5+AdamW+SE_Block+GDC	85.1	71.0	78.0	48.1	44.5	23.7	77.5
Retina Net	35.0	59.0	25.0	-	-	-	43.9
ATSS	51.0	50.0	32.0	-	-	-	50.5
Ours	85.7	72.8	79.8	51.6	75.6	9.8	78.6

**Table 6:** Summary of YOLOv8-based tomato disease detection studies.

Paper Author	Objective	Dataset Used	Performance Metrics	Integration into Robotic System
Hafedh Mahmoud Zayani et al. [27]	Tomato disease detection using YOLOv8	Custom dataset of tomato diseases (150 images per class, 19 classes)	mAP = 98%, F1 score = 97%	No direct integration described; potential for robotic application in tomato disease management
Wang Yonggui and He Jing [29]	Improvement in tomato disease detection with YOLOv8	Custom dataset for tomato diseases with 150 images per class	Improved mAP compared with original YOLOv8; exact metrics not specified	No direct integration described; potential for improvement in tomato farming operations
Arjun K et al. [30]	Pest detection and disease categorization in tomato crops using YOLOv8	Custom dataset of tomato pests and diseases	mAP = 92.5%; precision and recall not provided	Potential integration in robotic systems for pest and disease identification in tomato crops
Md. Shahriar Zaman Abid et al. [28]	Bangladeshi crop leaf disease detection using YOLOv8	Self-curated dataset of 2850 images (19 classes, 150 images per class)	mAP = 98%, F1 score = 97%	No specific robotic integration; applicable in field monitoring systems for disease management
Xuewei Wang and Jun Liu [31]	Vegetable disease detection using an improved YOLOv8 algorithm in greenhouse plant environment	Self-built vegetable disease dataset with 40,000 images from various vegetable types (Tomato, Cucumber, and Eggplant)	mAP = 92.91%, FPS = 271.07, and precision = 92.72%	Designed for use in greenhouse environments with potential for integration into automated agricultural systems
Mohammed Himeur and Abdelouahab Hassam [32]	Tomato disease detection using YOLOv8 with improved architecture for better accuracy	Dataset with 3400+ images (2800 for training, 400 for validation)	Precision = 82.43%, recall = 82.62%, and mAP@50 = 84.8%	Suitable for real-time crop monitoring systems
Ours	Optimizing robotic spraying for sustainable agriculture with an improved YOLOv8 algorithm for pesticide application on diseased tomato leaves	1500 tomato leaf images with 4 labels (Green Tomato, Downy Mildew, Powdery Mildew, and All)	Precision = 85.7%, recall = 72.8%, mAP@50 = 79.8%, mAP@50-95 = 51.6%, and F1 Score = 78.6%	Deployed on Spraying Robot LPE-260 for real-time, automated pesticide application

## Data Availability

The data presented in this study are available upon request from the corresponding author. The data are not publicly available due to privacy reasons.
